# Adjuvanted Protein Vaccines Boost RNA-Based Vaccines for Broader and More Potent Immune Responses

**DOI:** 10.3390/vaccines13080797

**Published:** 2025-07-28

**Authors:** Jiho Kim, Jenn Davis, Bryan Berube, Malcolm Duthie, Sean A. Gray, Darrick Carter

**Affiliations:** 1 PAI Life Sciences Inc., Seattle, WA 98102, USA; jiho.kim@pailifesciences.com (J.K.);; 2 HDT Bio Corp., Seattle, WA 98109, USA

**Keywords:** immunological adjuvants, RNA vaccines, COVID vaccines, pandemic preparedness

## Abstract

**Background/Objectives**: mRNA vaccines introduced during the COVID-19 pandemic were a significant step forward in the rapid development and deployment of vaccines in a global pandemic context. These vaccines showed good protective efficacy, but—due to limited breadth of the immune response—they required frequent boosters with manufactured spike sequences that often lagged behind the circulating strains. In order to enhance the breadth, durability, and magnitude of immune responses, we studied the effect of combining priming with an RNA vaccine technology with boosting with protein/adjuvant using a TLR4-agonist based adjuvant. **Methods**: Specifically, four proprietary adjuvants (EmT4^TM^, LiT4Q^TM^, MiT4^TM^, and AlT4^TM^) were investigated in combination with multiple modes of SARS-CoV-2 vaccination (protein, peptide, RNA) for their effectiveness in boosting antibody responses to SARS-CoV-2 spike protein in murine models. **Results**: Results showed significant improvement in immune response strength and breadth—especially against more distant SARS-CoV-2 variants such as Omicron—when adjuvants were used in combination with boosters following an RNA vaccine prime. **Conclusions**: The use of novel TLR4 adjuvants in combination with protein or RNA vaccinations presents a promising strategy for improving the efficacy of vaccines in the event of future pandemics, by leveraging rapid response using an RNA vaccine prime and following up with protein/adjuvant-based vaccines to enhance the breadth of immunity.

## 1. Introduction

The global spread of the human severe acute respiratory syndrome coronavirus 2 (SARS-CoV-2) started in 2019 and became a pandemic of unprecedented impact, affecting all forms of society including public health, economy, and social stability. As of August 2024, close to 800 million confirmed cases and 7 million deaths occurred as a result of the SARS-CoV-2 pandemic, and the true number of cases and deaths are likely much higher [1]. Although multiple highly effective vaccines that prevented severe illness and death were developed in a span of less than a year in what is seen as a herculean effort, there are still shortcomings in the fair distribution and effectiveness of the vaccine against the numerous variants that have arisen since the original variant. Africa received less than 2% of the global vaccine supply and issues like logistics, patent disputes, and export embargos enhanced the unequitable distribution of the vaccines to less wealthy countries [2]. The global impact of the SARS-CoV-2 pandemic is still very much apparent as of 2024 and will remain a seminary event in public health. As the urgency of the SARS-CoV-2 pandemic gradually fades into memory, however, the possibility of a future pandemic of similar magnitude and impact will be a priority for public health efforts for years to come [3].

The lessons learned in the development of vaccines against SARS-CoV-2 can and should also be used to plan for future pandemics, and an effective platform for the quick development, production, and distribution of vaccines is of paramount importance in such situations. Messenger RNA (mRNA)-based vaccines quickly became a dominant technology used against in the SARS-CoV-2 pandemic despite being a relatively newer technology. RNA-based vaccines were the first to be approved and distributed widely against COVID-19, with Pfizer-BioNTech’s Comirnaty^TM^ being authorized on an emergency basis in December 2020 followed by an approval by the US FDA in August 2021 [4]. The other primary RNA vaccine by Moderna (Spikevax) was also approved and widely distributed on a similar time frame as Comirnaty^TM^, with the two mRNA vaccines achieving a total of 3.3 billion doses produced as of January 2022 [5].

While slower to come to market, more traditional technologies such as antigen protein fragments and peptides could be used as “booster” immunogens either following or in combination with mRNA vaccination. Protein antigens have advantages in that current good manufacturing practice (cGMP)-compliant products can be produced with less stringent storage conditions, and such products, when lyophilized, can in some cases be stored at room temperature for years [6]. These characteristics make peptide and proteins attractive candidates for vaccine products to enhance RNA-based strategies in preparation for a future pandemic. Several subunit protein vaccine candidates have been developed and approved for COVID-19, including those by Novavax and Sanofi-GSK [7]. Distribution and uptake of these vaccines have not been as high as the RNA vaccines due to the delayed timing of approval and release and perceived inferiority in terms of efficacy. However, they provided an alternative to those sensitive to ingredients in mRNA vaccine formulations. Peptide vaccines have also been investigated and developed, with a candidate in Russia (EpiVacCorona) reaching Phase III clinical trials; while it was authorized in Russia, its efficacy has not been confirmed independently, and doubts remain regarding its rushed approval [8].

The introduction of RNA technology has certainly revolutionized the vaccine field, with the COVID-19 pandemic helping to thrust it to global prominence. RNA has several advantages compared to traditional vaccine technologies, including rapid production, deployment, and cost-efficiency, and the ability to easily modify the nucleotide sequence of the mRNA according to changes in the antigen (i.e., novel variants). The expression of the SARS-CoV-2 spike protein from mRNA vaccines in cells has been well-documented and investigated, with a robust immune response triggered upon administration of mRNA vaccines. However, several disadvantages of mRNA vaccines exist. First, the storage conditions for RNA vaccines are stringent, with most formulations requiring a cold chain maintained at −80 °C, which cannot be secured for low-resource and low-density environments. RNA is intrinsically a more fragile molecule compared to DNA or proteins and requires translocation into the cell cytosol in order to be translated into an effective antigen [9]. This necessitates its stabilization and cellular delivery through the use of nanoparticles or other carriers, and these particles may contain ingredients such as polyethylene glycol (PEG), which may trigger sensitivity in a small fraction of the population [10]. The most significant issue in the context of vaccine efficacy is the durability and breadth of the immune response. Humoral and cellular immunity fades within a span of months, and routine boosters are required to maintain responses. The drop in efficacy of RNA vaccines over time and the emergence of novel pathogen variants that escape the narrow breadth of immunity are problems for RNA vaccines that require solutions [11]. Our team has developed a self-amplifying RNA vaccine formulation making use of an alphavirus-based vector for RNA production, which attempts to address the durability of the response, as the protein expression from saRNA persists longer than that from mRNA. saRNA is complexed with a proprietary lipid inorganic nanoparticle (LION^TM^) carrier, which stabilizes its structure to allow for more flexible storage conditions—including at 4 °C, the temperature of a typical household refrigerator [12].

Another core concept of vaccines that has not been widely discussed in the SARS-CoV-2 pandemic has been the use of immunological adjuvants. Traditionally, these have been defined as formulations that augment the immune response by activating various immune sensors such as toll-like receptors (TLR) [13]. The presence of an adjuvant in a vaccine formulation thus serves to enhance the immunogenicity of the vaccine antigen, increasing the strength and the breadth of the immune response. Adjuvants are generally inexpensive, relatively straightforward to produce, and can be made ahead of time and stockpiled, enabling them to be implemented in a rapid and cost-effective manner for future pandemics [14]. Prime candidates for such adjuvants are those containing TLR agonists—particularly TLR4 agonists [15,16,17,18]—which have been widely studied and are in approved vaccine formulations such as the adjuvant AS01b in the shingles vaccine Shingrix^®^ [19]. Our team has developed a new generation of these TLR4 agonist-based formulations including a liposomal formulation of TLR4 agonist (LiT4Q^TM^), an emulsion (EmT4^TM^), an alum-adsorbed form (AlT4^TM^), and a micellar form of the adjuvant (MiT4^TM^) (Supplementary Table S1). Several of these adjuvant formulations have already been investigated in preclinical contexts in combination with an array of vaccine candidates [20,21].

In the study presented here, we investigated the potential of the new generation of TLR4 agonist adjuvants as formulated protein/adjuvant boosters to RNA immunization. The potential benefits of a mixed-mode vaccination regimen, i.e., to prime with a quick-response RNA vaccine and to boost with a cheaply and quickly made protein/adjuvant combination, could integrate the strengths of all modes involved [22]. A secondary objective was to investigate the combinations of spike proteins and peptide pools in combination with these adjuvants to boost the RNA vaccine response. We first selected well-performing adjuvant candidates out of the four adjuvants by vaccinating mice with an adjuvant + SARS-CoV-2 spike protein and subsequently measuring the antibody responses by ELISA and pseudovirus-neutralizing assays. Upon down-selecting three candidates, they were used in conjunction with RNA-based vaccine priming, as well as with peptides covering the length of the spike protein. The results indicated that all the selected adjuvants were able to boost the strength and breadth of the antibody response against several SARS-CoV-2 variants of significance—especially against the Omicron family. The highest efficacy was seen with the protein/adjuvant boost, while the results with the peptide/adjuvant boosting were ambiguous. Further, murine immunogenicity experiments confirmed the increased magnitude and breadth of the immune response when RNA regimens are combined with alternate immunogens (including proteins/peptides) adjuvanted with these formulations. The potential of mixed modes of immunization, combined with adjuvants, could serve as a quick and supplementary tool to combine with RNA vaccination in multiple infectious disease outbreaks and/or a future pandemic context.

## 2. Materials and Methods

### 2.1. Cell Lines

HEK293T cells were obtained from ATCC. HEK293T cells stably expressing human Angiotensin-Converting Enzyme 2 (HEK293T-hACE2) were obtained from BEI Resources (NR-52511). Cells were maintained in Dulbecco’s Modified Eagle Media (DMEM) with 10% fetal bovine serum, 1% penicillin/streptomycin, and 2 mM L-glutamine (cDMEM) and grown at 37 °C + 5% CO_2_.

HEK293F cells were obtained from Thermo-Fisher Scientific (Waltham, MA, USA). Cells were maintained in FreeStyle F17 Media supplemented with GlutaMax (Thermo-Fisher Scientific) and grown at 37 °C + 8% CO_2_.

#### Production of SARS-CoV-2 Immunogens

The full-length SARS-CoV-2 spike protein was produced by using an expression-optimized (and 6 × His-tagged) plasmid in pCMV vector for expression in HEK-293F cells (Thermo-Fisher Scientific). Briefly, cells were incubated in suspension before transfection using Lipofectamine 3000 (Thermo-Fisher Scientific) and allowed to produce protein for 48 h before harvesting the supernatant. The supernatants were run through nickel affinity columns and eluted using increasing concentrations of imidazole for purification. The purity and stability of the protein was confirmed by reducing agarose gel electrophoresis and visualization of one dominant band at approximately 78 kDa. Commercially available SARS-CoV-2 spike receptor binding domains (RBD) were procured from Sino Biological (Wayne, PA, USA).

SARS-CoV-2 peptides were produced by Thermo-Fisher (Pierce) Scientific. The peptides were each 49 amino acids long, with 25 amino acid overlaps covering the entire length SARS-CoV-2 D614G spike RBD, with cysteines removed to prevent disulfide bond structures, thus resulting in a total of 11 peptides covering a total of 223 amino acids. The peptides were produced using Fmoc solid-phase synthesis and reverse-phase high-performance liquid chromatography (HPLC). The quality and identity were verified by mass spectrometry and HPLC.

Self-amplifying RNA (saRNA) was produced at HDT Bio Corp. (Seattle, WA, USA) using a Venezuelan equine encephalitis virus-based platform. RNA was produced and analyzed following previously published protocols [12,23]. Briefly, RNA was transcribed off a plasmid containing the SARS-CoV-2 D614G spike protein using T7 RNA polymerase. RNA was stored at −80 °C in nuclease-free water before formulation with HDT Bio’s proprietary LION^TM^ carrier formulation prior to injection.

### 2.2. Adjuvant Production

Adjuvant formulations as presented in the paper were manufactured at PAI Life Sciences (Seattle, WA, USA). 3D-(6-acyl) PHAD^®^, Alhydrogel^TM^, phosphotidylcholines, and cholesterol were purchased from Avanti Polar Lipids, Inc. (Birmingham, AL, USA), part of Croda International Plc (Alabaster, AL, USA). Pharmacopeial grade solvents and surfactants were from Sigma/Aldrich (Burlington, MA, USA).

EmT4^TM^ was produced by making an oil phase, where squalene was sonicated with alpha-tocopherol at a 200:1 volume to mass ratio until dissolved; egg phosphotidylcholines (5:1 mass/mass) and the TLR4 agonist were added. This phase was homogenized by sonication in a sonicating bath at 60 °C for at least 1 h until completely homogenized. In parallel, 25 mM ammonium phosphate buffer was prepared (pH 5.4) by mixing mono- and di-basic ammonium phosphate in water for injection. Glycerol and Pluronic^®^ F-68 were added, and the components were mixed by stirring. Finally, the oil phase and aqueous phase were mixed using a Silverson Mixer for 10 min to blend the phases. The blended phase was then emulsified at 30,000 psi using a Microfluidics M110 microfluidizer. The final EmT4^TM^ was sterile filtered and vialed. The product was then stored at 2–8 °C until use.

LitT4Q^TM^ was produced by adding 3D-(6-acyl) PHAD to a 4:1 DOPC to cholesterol (mass/mass) mixture in chloroform in a flask and evaporated in the hood. This thin film was hydrated with 10 mM phosphate buffer pH = 6.1 to a concentration of 5 mg/mL DOPC, 1.25 mg/mL cholesterol, and 0.1 mg/mL 3D-(6-acyl) PHAD and sonicated in a water bath until the particle size reached 70–130 nm by dynamic light scattering (DLS). QS21 was solubilized into the mixture at 0.25 mg/mL and stirred at room temperature for at least 45 min prior to sterile filtration using a 0.22 μm filter.

The alum-adsorbed TLR4 agonist AlT4^TM^ was produced in a two-step process: First, a micellar suspension of the TLR4 agonist was produced (MiT4^TM^) by adding 3D-(6-acyl) PHAD to a flask, which was then combined with 1,2-dipalmitoyl-sn-glycero-3-phosphocholine (DPPC) and dissolved in chloroform. The chloroform was then evaporated, water for injection was added, and the mixture placed in a sonicating bath at 60 °C. Once the particle size averaged approximately 120 nm by DLS, the MiT4^TM^ was sterile-filtered. This suspension was then adsorbed to the surface of Alhydrogel^TM^ by mixing with water washed Alhydrogel at room temperature. Once combined, the AlT4^TM^ adjuvant was aliquoted into single use vials and capped for storage at 2–8 °C.

### 2.3. Animal Experiments

Female C57BL/6 mice were acquired from Charles River Laboratories at 6–8 weeks of age. Mice were anesthetized with isoflurane (2%) and checked for unconsciousness before injections. Formulations containing the immunogen and/or adjuvant were injected intramuscularly into both the right and left hind flank of with an injection volume of 50 µL per side. Adjuvants and antigens were mixed together and vortexed approximately 30 min prior to injection and vortexed again immediately prior to injection. Total doses of each mode per injection were as follows: full-length spike protein, 5 µg; saRNA, 1 µg; peptide pool, 5 µg (combined peptide dose). Blood was drawn at the indicated time points in serum collector tubes, and the serum was collected by centrifuging the blood samples at 3000× *g* for 10 min and collecting the resulting supernatant. The sera were subsequently stored at −80 °C until thawed for assays. Upon reaching the end of the experiment at the last indicated time point, the mice were euthanized by CO_2_ inhalation, followed by secondary cervical dislocation.

All animal studies were carried out in compliance with the University of Washington guidelines for animal experiments in line with an IACUC protocol, established by the University of Washington Department of Comparative Medicine.

### 2.4. Enzyme-Linked Immunosorbent Assay (ELISA)

Corning high binding 384-well plates (Corning, NY, USA) were coated with the indicated SARS-CoV-2 variant spike protein (PAI Life Sciences, Seattle, WA, USA, and Sino Biological, Wayne, PA, USA; Supplementary Table S2) in carbonate buffer. The coated plates were blocked prior to the addition of the diluted serum samples. The mouse anti-SARS-CoV-2 spike activity was detected using a HRP label Goat anti-Mouse IgG (H+L) polyclonal antibody (Southern Biotech, Birmingham, AL, USA) and visualized with acidified TMB (SeraCare, Milford, MA, USA). The reaction was read out at 450 nm, and the activity was analyzed using XLFit (IDBS, Woking, UK) analysis. Endpoint titers were calculated by fitting a sigmoidal 4-parameter curve to the data, and the cutoff was defined as the OD450 average of the negative (unimmunized) sera samples + 6*SD of the negative (unimmunized) sera samples.

Isotype ELISAs were carried out identically to the above, except for the use of the following secondary antibodies: goat anti-mouse IgG1 Human-adsorbed-HRP, goat anti-mouse IgG2a/IgG2c-HRP, or goat anti-mouse IgG3 Human-adsorbed HRP (Southern Biotech, Birmingham, AL, USA).

### 2.5. Pseudovirus-Neutralization Assays

SARS-CoV-2 Spike pseudoviruses were produced by co-transfecting HEK293T cells with plasmids in the SARS-CoV-2 Spike-pseudotyped lentiviral particle kit (BEI Resources; NR-52948) and a plasmid encoding the SARS-CoV-2 Spike protein (Wuhan-Hu-1; Genbank: NC 045512) containing a D614G mutation. Taking this base plasmid, the sequence was modified and cloned to contain the BA.1 pseudovirus sequences to produce the BA.1 pseudovirus. Cells were incubated for 48–56 h, and the supernatants were collected and filtered with a 0.45 µm filter. Aliquots were prepared and stored at −80 °C until use.

Pseudovirus neutralization assays were carried out as previously described [24]. Briefly, HEK293T-ACE2 cells were plated in a poly-L-lysine-coated 96-well plate and incubated for 24 h at 37 °C + 5% CO_2_. Serum samples were diluted in cDMEM in a 10-point half-log dilution series starting at a 1:50 dilution. Serum dilutions were mixed 1:1 with diluted pseudovirus (1:100 in cDMEM for D614G, 1:200 in cDMEM for Omicron (BA.1)) and incubated for 1 h at 37 °C + 5% CO_2_ before addition to cells. After 48 h, the media was removed, and Bright-Glo luciferase reagent (Promega Corporation, Madison, WI, USA) added to the cells. The luminescence was measured on a i3x spectrophotometer (Molecular Devices, San Jose, CA, USA). The luminescence values were plotted against the serum dilutions, and dose–response curves were generated using a 5-parameter fit. IC50 values were calculated as the reciprocal serum dilution that inhibited the luciferase signal by 50% relative to the virus-only wells.

### 2.6. Statistical Analyses

All statistical analyses and graphing of data were performed using GraphPad Prism 10.4 (GraphPad Software, San Diego, CA, USA). Timepoints were compared between vaccinated groups and controls using two-way ANOVA with Tukey’s multiple comparisons test. Significant differences are labeled accordingly in the figures, where * *p* < 0.05, ** *p* < 0.01, *** *p* < 0.001, and **** *p* < 0.0001.

If groups of 0 values (or below limit of detection) were present, groupwise comparisons were carried out using pairwise multiple *t*-tests of the log values of titers or IC50s corrected for the false-discovery rate, with the significance defined as q < 0.01, with graphs labelled accordingly with the significance of * *p* < 0.05, ** *p* < 0.01, *** *p* < 0.001, and **** *p* < 0.0001.

To calculate the total sample size of mice required per group, Fisher’s exact test (2-sided) was used as a basis, assuming the minimum detectable difference in response proportions P_E_ (experimental) and P_C_ (control) with 80% power and a type I error rate of 0.05. If P_C_ = 0 (no response in control animals), and P_E_ = 0.5 (any measurable immune response in 50% of the experimental animals), the number of animals needed per group is 10. Data obtained from the studies outlined above were statistically analyzed by a Fisher’s exact test for this primary analysis. Significance as indicated by *p*-values of less than 0.05. A smaller number of mice (n = 5) was used for the initial screening experiment due to a larger expected effect (i.e., P_E_ = 0.7) and the exploratory nature of the experiment. Data collection and analysis were performed by blinded personnel distinct from personnel handling and administering treatment to mice.

## 3. Results

### 3.1. Adjuvants in Combination with Spike Protein Enhance the Breadth and Magnitude of the Immune Response

**Figure 1:**
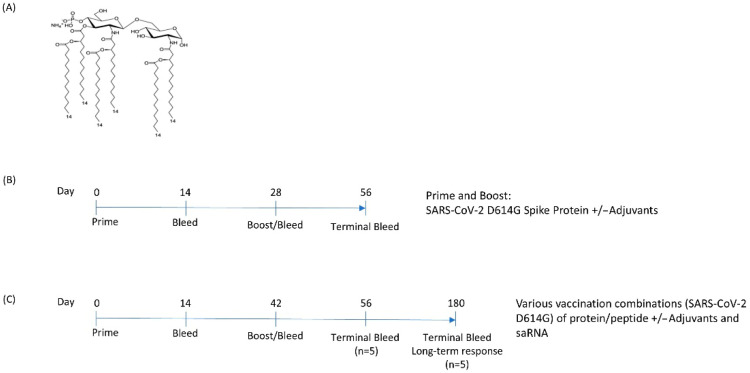
Premises for the experiment making use of proprietary TLR-4 agonist adjuvants. (**A**) Chemical structure of the TLR4 agonist used in next-generation adjuvant formulations LiT4Q^TM^, EmT4^TM^, MiT4^TM^, and AlT4^TM^; (**B**) mouse animal experiment timeline for initial experiment to down-select the best-performing adjuvants; (**C**) mouse animal experiment timeline for second set of experiments investigating multimodal vaccination involving adjuvants and a combination of RNA, protein, or peptide vaccines.

Novel adjuvants were formulated with a novel TLR4 agonist 3D-(6-acyl) PHAD^®^, an MPL-A (monophosporyl-lipid A) analog as the active ingredient (Figure 1A). To assess the efficacy of all four adjuvant formulations and to narrow down the number to be tested multimodally, mice were injected with a combination of spike protein and adjuvant with a timeline according to Table 1 and Figure 1B.

The mice tolerated the treatment well, and no significant safety or health abnormalities were noted for the duration of the experiment. The serum samples collected from the mice were tested for antibody responses against spike proteins from various SARS-CoV2 variants by ELISA as outlined in Supplementary Table S2.

**Figure 2:**
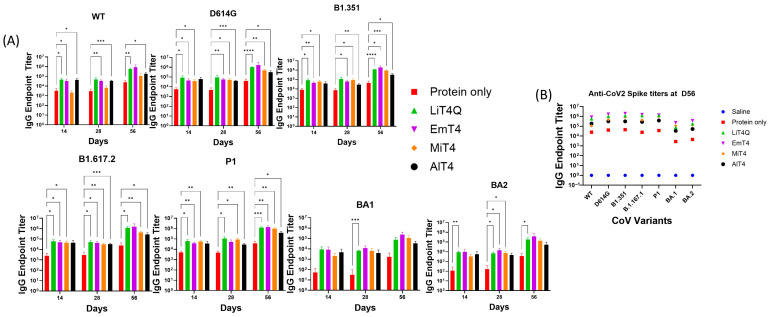
Anti-SARS-CoV-2 antibody responses in mice immunized with spike protein + adjuvant. Mice were immunized with a SARS-CoV-2 D614G protein in combination with a specified adjuvant at days 0 (prime) and 28 (boost). (**A**) Anti-SARS-CoV-2 IgG endpoint titer values are presented in the graphs, against the indicated SARS-CoV-2 variant spike protein. The bars represent the mean values of endpoint titers with standard error bars. (**B**) Summary mean values of anti-SARS-CoV-2 titers against each variant are shown at day 56, the last time point when serum samples were collected. Table 2 shows the numeric values and standard error of the endpoint titers of antibody isotypes IgG1, IgG2c, IgG3, and total IgG against the SARS-CoV-2 D614G protein generated in response to immunization at day 56 post-immunization. * *p* < 0.05, ** *p* < 0.01, *** *p* < 0.001, and **** *p* < 0.0001.

The response in the unadjuvanted D614G protein group was highest at termination (Figure 2A,B, day 56), with an anti-CoV2 spike IgG endpoint titer of 41,047 against the D614G spike and 4486 against the Omicron BA.1 variant. The responses were boosted by all adjuvanted formulations at the time of termination for all variant spikes tested—including the more distant Omicron (BA.1 and BA.2) variants. All adjuvants demonstrated boosted IgG responses against the various mutant spike proteins tested (Figure 2A), with EmT4^TM^ and LiT4Q^TM^ generally demonstrating the largest increases in IgG endpoint titers in comparison to the unadjuvanted group, with this difference becoming the clearest at day 56 (Figure 2B). MiT4^TM^- and AlT4^TM^-boosted groups also showed broad increases in endpoint titers against all variant spikes tested including BA.1 and BA.2 but were ranked slightly behind EmT4^TM^ and LiT4Q^TM^ (Figure 2A). The antibody isotype analysis of immunized mice sera at day 56 post-immunization indicated a predominantly IgG1 response, with all four adjuvants generating higher IgG1 responses in comparison to the unadjuvanted protein group (Table 2). For IgG2c and IgG3 responses, all adjuvants except AlT4 generated higher responses compared to the unadjuvanted protein (Table 2). All adjuvants demonstrated a significantly increased strength of response against more closely related variants (D614G, B1.1.351, B1.617.2, P1) as compared to the more distant Omicron family (BA.1, BA.2) (Figure 2).

**Figure 3:**
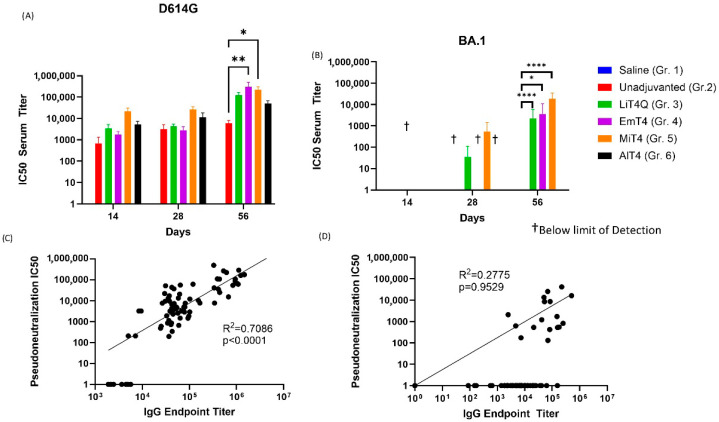
SARS-CoV-2 pseudovirus neutralization post-immunization with SARS-CoV-2 spike protein and adjuvant. After a prime and boost of SARS-CoV-2 D614G spike protein + indicated adjuvant on days 0 and 28, respectively, serum samples were taken at days 14, 28, and 56. Serum samples were tested for pseudovirus neutralization capabilities, with the mean IC50 values presented against (**A**) D614G spike- and (**B**) Omicron BA.1 spike-expressing pseudoviruses. Bars indicate mean IC50 values as calculated according to the methods. Saline (Group 1) resulted in all values below the limit of detection for pseudoneutralization. Pseudoneutralization IC50 values were correlated with the IgG endpoint titers, as presented in Figure 2, for (**C**) D614G and (**D**) Omicron BA.1 spike protein or pseudovirus, respectively. All pairs of pseudovirus neutralization IC50 and IgG endpoint titers were plotted and fit to a log–log linear regression to determine the goodness of fit and significance of correlation. † All values in group were below limit of detection. * *p* < 0.05, ** *p* < 0.01, **** *p* < 0.0001.

Mouse sera were tested for their capability to neutralize SARS-CoV-2 pseudoviruses expressing either the D614G spike or Omicron (BA.1) spike on their surface (Figure 3). A luciferase-based pseudovirus neutralization assay indicated a low neutralizing capability in the unadjuvanted spike-only group (Group #2) even after boost, but the addition of an adjuvant resulted in a boost to the neutralizing response analogous to the anti-spike antibody responses seen in the ELISAs. The highest pseudovirus neutralizations were in group #4, EmT4^TM^, followed by MiT4^TM^ and LiT4Q^TM^ (Figure 3A); with the exception of AlT4^TM^ (Group #6), all adjuvant-boosted groups performed similarly. EmT4 and MiT4 both showed significantly higher titers compared to the unadjuvanted group at day 56 (Figure 3A).

This pattern mostly extended to Omicron (BA.1) pseudovirus, but the response showed a slower development over time when compared to the D614G spike. Neutralizing titers appeared by day 56 at termination (8 weeks) post-prime, and at this time point, all groups except AlT4 showed discernible pseudovirus neutralization titers, in comparison to the unadjuvanted group, which showed no detectable neutralization. All adjuvants except AlT4 demonstrated a degree of neutralization by D56, and significant differences were noted at this time point between all adjuvants (except AlT4^TM^) and the unadjuvanted group (Figure 3B). IgG endpoint titers and virus neutralization IC50s were moderately correlated when comparing the results against D614G protein or pseudovirus, respectively, with a linear log–log R^2^ of 0.7086 (*p* < 0.0001). (Figure 3C). However, a linear correlation was not observed when comparing the Omicron BA.1 IgG endpoint titers and pseudovirus IC50 titers, likely due to multiple instances of 0 (no detectable neutralization) IC50 pseudoneutralization (Figure 3D). Thus, multiple adjuvant formulations of the TLR4-agonist in combination with a spike protein immunization resulted in both increased strength (by increased IgG titers and pseudovirus neutralization) and increased breadth (by responses against multiple variants) of the immune response against SARS-CoV-2 compared to an unadjuvanted protein immunization.

### 3.2. Key Adjuvants in Combination with mRNA and Peptides to Boost the Magnitude of the Immune Response

From the first set of experiments, EmT4^TM^, LiT4Q^TM^, and MiT4^TM^ were established as the most promising adjuvant candidates to be used going forward, considering both the ELISA and the pseudovirus neutralization results. Multiple vaccination modes (protein, peptide, saRNA) were introduced into a second phase of the experiment (Table 3). New additional modes included a self-amplifying RNA (saRNA) vaccine candidate encoding D614G spike developed by HDT Bio, and peptide pools covering the SARS-CoV2 D614G spike protein with overlaps. Mice were primed and boosted according to a similar schedule as in the first experiment, with the boost taking place at day 42 post-prime—an optimized duration for saRNA vaccinations (Figure 1C). Mouse sera were collected at 0, 14, and 42 days. Half the mice of each group were terminated at day 56, and the other half were terminated at day 180 to gauge both short-term and long-term responses. Serum samples were collected at termination for antibody and pseudovirus neutralization titers.

**Figure 4:**
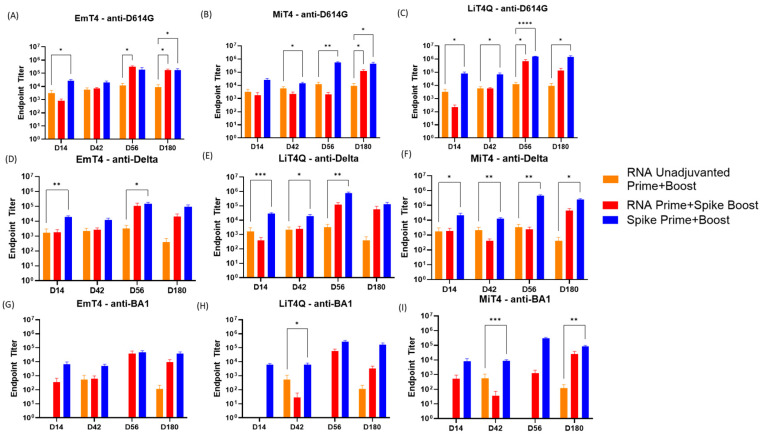
Anti-SARS-CoV-2 antibody response in mice immunized with multimodal spike protein and/or RNA in combination with adjuvant. Mice were immunized with the indicated regimen at days 0 (prime) and 42 (boost). Serum was tested for IgG responses against (**A**–**C**) D614G, (**D**–**F**) Delta P1, or (**G**–**I**) Omicron BA1 spike protein in an anti-SARS-CoV-2 IgG ELISA. Bars indicate mean IgG endpoint titer values with standard error bars. * *p* < 0.05, ** *p* < 0.01, *** *p* < 0.001, and **** *p* < 0.0001.

All mice involved in the second set of experiments displayed no significant safety symptoms and well tolerated the vaccination regimens involved in the duration of the experiment. ELISA results indicated the responses increased by day 42, with the strongest responses occurring in groups that received a protein-adjuvant boost and prime (Blue bars, Figure 4A–C). The comparison standard was taken to be the unadjuvanted RNA prime and boost group (Figure 4, Orange Bars, Group 3). Of these, the highest endpoint titers were reached by the group that received the D614G Spike protein plus LiT4Q^TM^ combination prime and boost (Figure 4B, Averaging 1,383,819 anti-D614G titers at D180). It was notable that the protein/adjuvant prime and boost groups all had durable responses lasting the entire duration of the study at 6 months regardless of the adjuvant received. Groups receiving a peptide/adjuvant boost and prime (Supplementary Figure S1A–C) were the least effective in eliciting a response, although responses were detected by day 42. However, these responses were not durable, as titers either decreased or were undetectable again by day 180. The unadjuvanted RNA prime and boost (Figure 4, Orange Bars, Group 3) resulted in a moderate response by day 42 which lasted until day 180; however, the magnitude of the response was noticeably lower than that of the adjuvanted groups, significantly so when compared to either the RNA Prime + Spike Protein boost or the homologous Spike Prime + Boost. RNA primes boosted by protein/adjuvant (Red Bars, Groups 4, 5, 6) all resulted in enhanced responses at both the day 42 and day 180 time points, with the highest endpoints occurring in the group boosted by D614G Protein + LiT4Q^TM^ (Figure 4B; Group 5, average 134,383 at day 180). RNA prime groups boosted by peptide/adjuvant resulted in minimal responses at both day 42 and day 180, with the exception of the group boosted by D614G Spike peptides plus MiT4, which reached an average titer of 750,893 by day 56 (Supplementary Figure S1A). However, this response did not last, as titers decreased by day 180. No groups involving peptide primes and/or boosts showed significant differences from the RNA prime + boost group for all antigens involved (Supplementary Figure S1A–C).

Compared to the baseline of RNA prime and boost which could be seen as comparable to current mRNA vaccine regimens, the presence of an adjuvanted alternate immunogen (i.e., spike protein) boosted the response significantly, indicating that existing RNA vaccination regimens could potentially be boosted further for an enhanced magnitude and duration of the immune response, which could last at least 180 days in immunized mice.

### 3.3. Key Adjuvants in Combination with RNA and Peptides to Boost the Breadth of the Immune Response

We then examined the breadth of the immune response triggered by these immunization regimens by exploring the antibody response by ELISA against two other SARS-CoV-2 variants, Delta (B1.617) and Omicron (BA.1) (Figure 4D–I). As expected, responses against these variants were diminished when compared to the original immunogen (D614G), and this effect was especially marked for the BA.1 variant, which is a more distant variant from D614G than Delta. The results were broadly similar to what was seen with the D614G response, with the protein-adjuvanted groups showing the highest endpoint titers found by total IgG ELISA. Again, the highest titers were seen in the protein/adjuvant prime and boost groups, with the protein/LiT4Q^TM^ group resulting in the highest titers against both Delta and Omicron variant spike proteins. It is notable that while the RNA prime and boost resulted in a lower response against BA1 (Figure 4G–I, Orange Bars, Group 3), the addition of LiT4Q or MiT4 adjuvant and protein boost resulted in significant increases in titer response; however, significant increases were only observed with a homologous adjuvanted spike protein prime and boost with LiT4Q or MiT4 (Figure 4H,I). These results suggest that the inclusion of an adjuvanted protein boost, in addition to an RNA prime, resulted in increased immune responses against more distant SARS-CoV-2 variants such as Omicron.

### 3.4. Pseudovirus Neutralization Capabilities of Prime + Boost Regimens

**Figure 5:**
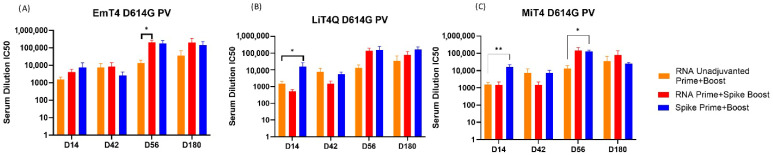
SARS-CoV-2 pseudovirus neutralization post-immunization with multimodal SARS-CoV-2 antigen and adjuvant. Serum samples were collected from groups immunized with combinations including D614G spike protein in combination with (**A**) EmT4^TM^, (**B**) LiT4Q^TM^, and (**C**) MiT4^TM^ as indicated in legend. Serum samples were tested for pseudovirus neutralization capabilities, with the mean IC50 values presented against (**A**–**C**) a D614G spike-expressing pseudovirus. * *p* < 0.05, ** *p* < 0.01.

To further gauge the humoral response against SARS-CoV-2, pseudovirus neutralization assays were performed against SARS-CoV-2 D614G pseudoviruses. Supporting the previously found ELISA results, the boosting capability of a protein + adjuvant combination was still apparent in combination with an RNA prime (Figure 5A). The best performing groups were protein + adjuvant prime and boost (Groups 7, 8, 9) and RNA prime + protein/adjuvant boost (Groups 4, 5, 6). In contrast to the ELISA results, there was no clearly superior regimen in this case, as all of the aforementioned groups were more or less comparable in IC50 neutralization values. The baseline group of RNA prime and boost (Figure 5A–C, Orange Bars, Group 3) trended lower neutralization capability in comparison to the adjuvanted boost groups. Regimens involving adjuvanted peptide resulted in negligible neutralization (Supplementary Figure S1D), when compared to RNA-only prime and boost. 

Out of the three adjuvants tested, EmT4^TM^ and LiT4Q^TM^ had comparable results, whereas MiT4^TM^ resulted in slightly lower IC50 values although it demonstrated the only significant increase in neutralization compared to unadjuvanted RNA at day 56 when used in combination with a homologous protein prime and boost (Figure 5C). Overall, the protein/adjuvant prime and boost regimen remained one of the best performing groups in terms of strength and duration, corroborating the ELISA data.

The results obtained from SARS-CoV-2 D614G pseudovirus indicated mostly similar results to anti-SARS-CoV-2 spike protein ELISAs. Thus, this further indicated that the adjuvanted protein could boost the magnitude and duration of the immune response against SARS-CoV-2 spike protein and pseudovirus.

## 4. Discussion

The COVID-19 pandemic revealed both the strengths and weaknesses of RNA based vaccine technologies, with rapid deployment being a key strength [25]. Limitations in the durability and breadth of the immune response was a challenge as multiple changes to the spike protein of the circulating virus required frequent boosters and changes to the vaccine antigen being manufactured [26]. Future pandemics may involve similar pathogens, requiring a vaccine development response incorporating the strengths of multiple modes of vaccination. Here, we present studies in mice on combining a self-amplifying RNA vaccine prime with a boost using various modes of antigens combined with TLR4-based adjuvant formulations. In this study, the incorporation of TLR4-agonist adjuvants, particularly EmT4^TM^, LiT4Q^TM^, and MiT4^TM^, demonstrated a clear enhancement in the immune response’s magnitude and breadth compared to RNA vaccines alone.

The combination of saRNA priming followed by protein/adjuvant boosting appears to be a promising approach to achieving both rapid immunity and lasting protection. Specifically, the use of LiT4Q^TM^ and EmT4^TM^ was associated with improved antibody responses and pseudovirus neutralization, suggesting that these adjuvanted formulations can help overcome some of the limitations of RNA-alone vaccine strategies: the improved immune responses against more distant variants like Omicron BA.1 indicate that this combination approach may help to generate a broader spectrum of immunity, which is crucial for addressing emerging variants. As seen in the isotype results, more IgG2 was produced in response to the adjuvanted formulations that also were better at neutralizing the virus. This skewing could explain the ranking of the adjuvants in terms of which ones were most potent: those that enabled more Th1 bias also neutralized better. There is not a direct correlation between titer and neutralization. As seen with other viruses, the quality of the response is important. It also may be a marker of T cell help induced by the TLR-based adjuvants. While not measured as part of these studies, it is likely that T cell help—enhanced by the adjuvant formulations—was important for the high-quality antibody response to the protein antigens. This T cell help—or lack thereof potentially in peptide vaccines—may also explain why the peptide-only formulations did not provide a high-quality response. These peptide pools covering the spike protein were investigated as a mode of heterologous boosting but did not demonstrate efficacy to the extent that was observed with spike protein. This also may corroborate the lack of real-world success in the development of an approved SARS-CoV-2 peptide vaccine. The results as observed indicate that a multimodal boosting of mRNA vaccines could be especially beneficial in providing sustained immune protection without the need for frequent booster doses, as has been the case with mRNA-only vaccines [27].

## 5. Conclusions

One of the key takeaways from this study is the potential for combining the rapid response capabilities of RNA-based vaccines with the broader and more durable immune responses achievable through protein/adjuvant technologies [28,29,30]. This approach could be vital for future pandemic preparedness, offering a two-pronged strategy that allows for both an immediate response to an emerging threat and a subsequent more durable immunity boost once adjuvant-based formulations are available. The findings of this study support the notion that an integrated approach of adjuvanted protein vaccine technologies [28,29,30] with RNA vaccines could serve as a foundation for next-generation vaccine design, ensuring both rapid deployment of the RNA and long-term efficacy against a wide array of variants provided by the protein/adjuvant boost. Indeed, the use of heterologous vaccines (combinations of protein, viral vector, and/or mRNA) has been extensively documented during the SARS-CoV-2 pandemic [31,32,33], and such regimens could potentially leverage multiple strengths of the various modes. This study only establishes the possibility of using this strategy in a future pandemic context with preliminary murine data supporting the use of the combinations explored, and while mice have limitations as a model, we feel the results here, when seen in the context of human field results on short lived and very narrow responses with RNA vaccines, demonstrate that the findings will translate to humans. Further investigation in human contexts and the mechanisms behind the adjuvants’ efficacy and moving this strategy further into human clinical trials could enhance global readiness against another potentially devastating pandemic.

## Figures and Tables

**Table 1:** Study design animal groupings for adjuvant down-selection.

Group	n	Prime (D0)		Boost (D28)	
		Antigen	Adjuvant	Antigen	Adjuvant
1	5	Saline	None	Saline	none
2	5	5 µg D614G Spike Protein	None	5 µg D614G Spike Protein	none
3	5		LiT4Q^TM^		LiT4Q^TM^
4	5		EmT4^TM^		EmT4^TM^
5	5		MiT4^TM^		MiT4^TM^
6	5		AlT4^TM^		AlT4^TM^

**Table 2:** Antibody isotype ELISA endpoint titers at D56 for adjuvant down-selection experiment.

Isotype		Immunization Adjuvant					
		Control (PBS)	Protein Only	+LiT4Q^TM^	+EmT4^TM^	+MiT4^TM^	+AlT4^TM^
IgG1	Endpoint titer	0	98,858	427,271	256,998	185,521	949,823
	Standard error	0	21,270	78,937	31,741	50,874	144,934
	*p*-value vs. Protein only	-	-	* 0.0327	** 0.0078	0.3681	* 0.0157
IgG2a	Endpoint titer	0	0	6047	6652	3670	0
	Standard error	0	0	920.7	878.4	396.0	0
	*p*-value vs. Protein only	-	-	†	†	†	†
IgG3	Endpoint titer	0	468	14,660	5930	3153	117
	Standard error	0	467	3850	2388	1323	116
	*p*-value vs. Protein only	-	-	0.0681	0.1326	0.3630	0.8888
Overall IgG	Endpoint titer	0	41,407	1,022,269	1,708,718	469,938	320,410
	Standard error	0	9521	37,003	669,855	48,279	52,482
	*p*-value vs. Protein only	-	-	*** <0.0001	0.1714	** 0.0019	* 0.0149

* *p* < 0.05, ** *p* < 0.01, *** *p* < 0.001. *p*-value vs. protein only; † In the IgG2c analysis, all mice in the unadjuvanted protein group had no detectable IgG2c response, thus preventing a meaningful statistical comparison to the adjuvanted groups.

**Table 3:** Study design animal groupings for vaccination formulations.

Group	n	Prime (D0)		Boost (D42)	
		Antigen	Adjuvant	Antigen	Adjuvant
1a	5	Saline	None	None	EmT4^TM^
1b	5	Saline	None	None	LiT4^TM^
2	10	1 µg D614G saRNA	None	Saline	None
3	10	1 µg D614G saRNA	None	1 µg D614G saRNA	None
4	10	1 µg D614G saRNA	None	5 µg D614G Spike	EmT4^TM^
5	10	1 µg D614G saRNA	None	5 µg D614G Spike	LiT4Q^TM^
6	10	1 µg D614G saRNA	None	5 µg D614G Spike	MiT4^TM^
7	10	5 µg D614G Spike	EmT4^TM^	5 µg D614G Spike	EmT4^TM^
8	10	5 µg D614G Spike	LiT4Q^TM^	5 µg D614G Spike	LiT4Q^TM^
9	10	5 µg D614G Spike	MiT4^TM^	5 µg D614G Spike	MiT4^TM^
10	10	1 µg D614G saRNA	None	5 µg D614G Peptides	EmT4^TM^
11	10	5 µg D614G Peptides	EmT4^TM^	5 µg D614G Peptides	EmT4^TM^
12	10	1 µg D614G saRNA	LiT4Q^TM^	5 µg D614G Peptides	LiT4Q^TM^
13	10	5 µg D614G Peptides	LiT4Q^TM^	5 µg D614G Peptides	LiT4Q^TM^
14	10	1 µg D614G saRNA	MiT4^TM^	5 µg D614G Peptides	MiT4^TM^
15	10	5 µg D614G Peptides	MiT4^TM^	5 µg D614G Peptides	MiT4^TM^

## Data Availability

All authors have agreed to the conditions for publication set forth by the journal. Study data can be provided upon request.

## Supplementary Materials

The following supporting information can be downloaded at: https://www.mdpi.com/article/10.3390/vaccines13080797/s1, Figure S1: Testing peptides as a vaccination mode in combination with adjuvants; Table S1: Adjuvant Formulation Description; Table S2: Panel of SARS-CoV2 proteins; Table S3: Sequences of SARS-CoV-2 RBD Peptides.
