# Supplementary material for: Adjuvanted Protein Vaccines Boost RNA-Based Vaccines for Broader and More Potent Immune Responses
Source: Vaccines (Basel). 2025 Jul 28;13(8):797. doi: 10.3390/vaccines13080797 (PMC12389852; doi:10.3390/vaccines13080797)
Supplement: Supplementary file 1 [file vaccines-13-00797-s001.zip › vaccines-3753187-supplementary.pdf]

Supplementary Information

Supplementary Table S1. Adjuvant Formulation Description.

**Adjuvant**	**Comparable Adjuvant**	**Formulation**
LiT4Q	AS01b	3D(6-acyl)-PHAD + QS21 in liposome, ~100nm
EmT4	AS03, MF59, GLA-SE	3D(6-acyl)-PHAD in oil-in-water emulsion
MiT4	GLA-AF	3D(6-acyl)-PHAD in micelle
AlT4	AS04	3D(6-acyl)-PHAD adsorbed onto alum

Supplementary Table S2. Panel of SARS-CoV2 proteins.

#	Variant Identification		Source
	WHO	PANGO	
1	Wuhan Native	--	PAI Life Sciences
2	D614G	D614G	PAI Life Sciences
3	Alpha	B1.1.7	PAI Life Sciences
4	Beta	B1.351	PAI Life Sciences
5	Gamma	P1	Sino Biological
6	Delta	B1.617	Sino Biological
7	Omicron	BA.1	Sino Biological
8	Omicron	BA.2	Sino Biological

Supplementary Table S3. Sequences of SARS-CoV-2 RBD Peptides.

#	Amino Acid Sequence	Position # within SARS-CoV-2 Spike
1	RVQPTESIVRFPNITNLSPFGEVFNATRFASVYAWNRKRISNSVADYSV	338-386
2	FNATRFASVYAWNRKRISNSVADYSVLYNSASFSTFKSYGVSPTKLNDL	361-409
3	SVLYNSASFSTFKSYGVSPTKLNDLSFTNVYADSFVIRGDEVRQIAPGQ	385-433
4	LSFTNVYADSFVIRGDEVRQIAPGQTGKIADYNYKLPDDFTGSVIAWNS	409-457
5	QTGKIADYNYKLPDDFTGSVIAWNSNNLDSKVGGNYNYLYRLFRKSNLK	433-481
6	SNNLDSKVGGNYNYLYRLFRKSNLKPFERDISTEIYQAGSTPSNGVEGF	457-505
7	KPFERDISTEIYQAGSTPSNGVEGFNSYFPLQSYGFQPTNGVGYQPYRV	481-524
8	FNSYFPLQSYGFQPTNGVGYQPYRVVVLSFELLHAPATVSGPKKSTNLV	505-553
9	VVVLSFELLHAPATVSGPKKSTNLVKNKSVNF	529-560

Supplementary Figure S1. Testing peptides as a vaccination mode in combination with adjuvants – IgG and Pseudoneutralization Titers.

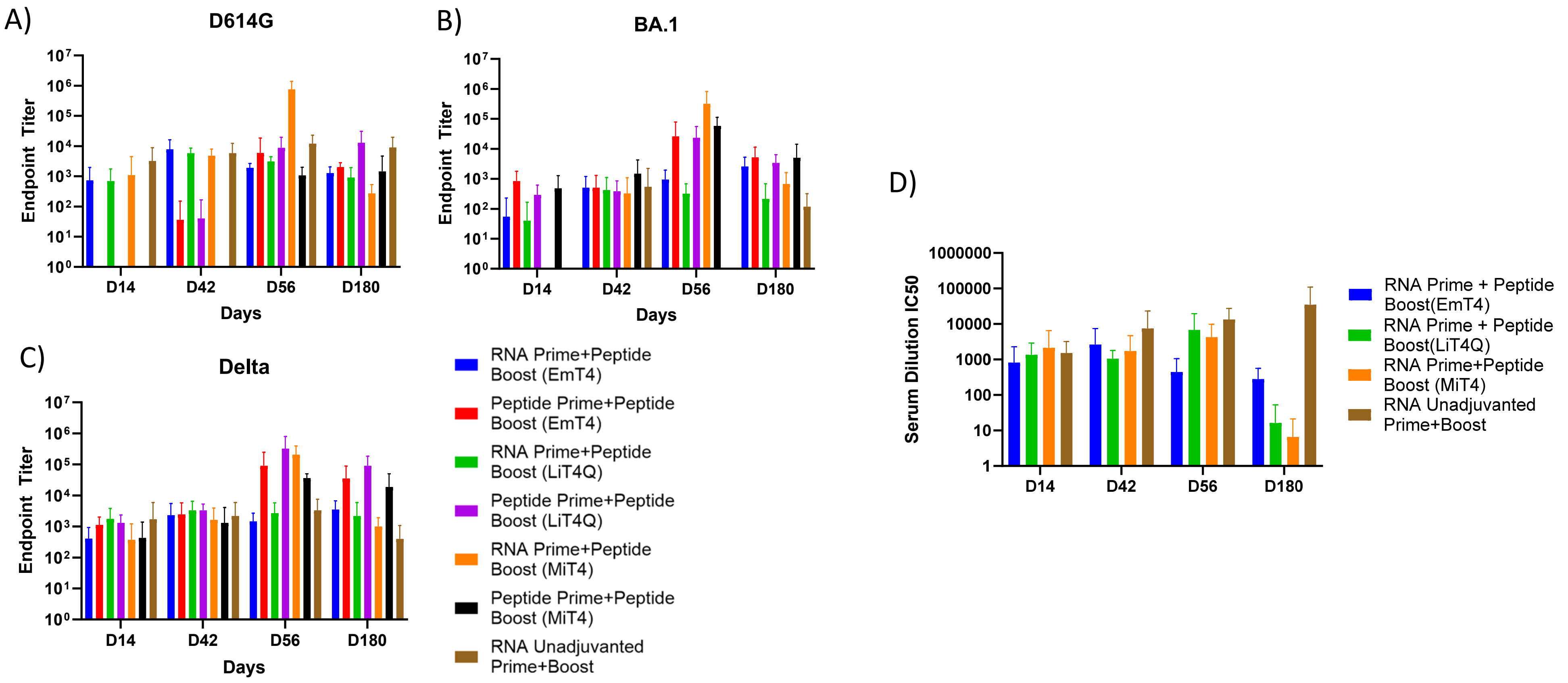

Supplementary Figure S1. Testing peptides as a vaccination mode in combination with adjuvants. Vaccination regimens involving peptide pools covering SARS-CoV-2 D614G spike protein according to Figure 1c were administered to C57BL/6 mice. Sera were collected at indicated time points and **A-C)** anti-SARS-CoV-2 spike protein IgG and **D)** D614G pseudovirus neutralization IC50 were obtained. The homologous unadjuvanted RNA prime and boost group (in brown) is shown as a comparable control group.
